# Comparison of In Vitro- and Chorioallantoic Membrane (CAM)-Culture Systems for Cryopreserved Medulla-Contained Human Ovarian Tissue

**DOI:** 10.1371/journal.pone.0032549

**Published:** 2012-03-30

**Authors:** Vladimir Isachenko, Peter Mallmann, Anna M. Petrunkina, Gohar Rahimi, Frank Nawroth, Katharina Hancke, Ricardo Felberbaum, Felicitas Genze, Ilija Damjanoski, Evgenia Isachenko

**Affiliations:** 1 Research Group for Reproductive Medicine and IVF-Laboratory, CAM-Xenotransplantation Group, Department of Obstetrics and Gynecology, Cologne University, Cologne, Germany; 2 Unit of Reproductive Medicine of Clinics, University of Veterinary Medicine, Hannover, Germany; 3 Laboratory of Flow Cytometry, Cambridge Institute for Medical Research, University of Cambridge, Cambridge, United Kingdom; 4 Department for Reproductive Medicine, Center for Endocrinology and Reproductive Medicine in Barkhof, Hamburg, Germany; 5 University Maternal Hospital, Department of Obstetrics and Gynecology, Ulm University, Ulm, Germany; 6 University Maternal Hospital Kempten, Department of Obstetrics and Gynecology, Ulm University, Kempten, Germany; 7 University Hospital for Urology, Ulm University, Ulm, Germany; National Cancer Center, Japan

## Abstract

At present, there are three ways to determine effectively the quality of the cryopreservation procedure using ovarian tissue before the re-implantation treatment: evaluation of follicles after post-thawing xenotransplantation to SCID mouse, in-vitro culture in a large volume of culture medium under constant agitation and culture on embryonic chorio-allantoic membrane within a hen's eggs. The aim of this study was to compare the two methods, culture in vitro and culture on embryonic chorioallantoic membrane (CAM) of cryopreserved human ovarian medulla-contained and medulla-free cortex. Ovarian fragments were divided into small pieces (1.5–2.0×1.0–1.2×0.8–1.5) of two types, cortex with medulla and medulla-free cortex, frozen, thawed and randomly divided into the following four groups. Group 1: medulla-free cortex cultured in vitro for 8 days in large volume of medium with mechanical agitation, Group 2: medulla-containing cortex cultured in vitro, Group 3: medulla-free cortex cultured in CAM-system for 5 days, Group 4: medulla-containing cortex cultured in CAM-system. The efficacy of the tissue culture was evaluated by the development of follicles and by intensiveness of angiogenesis in the tissue (von Willebrand factor and Desmin). For Group 1, 2, 3 and 4, respectively 85%, 85%, 87% and 84% of the follicles were morphologically normal (P>0.1). The immunohistochemical analysis showed that angiogenesis detected by von Willebrand factor was lower in groups 1 and 3 (medulla-free cortex). Neo-vascularisation (by Desmin) was observed only in ovarian tissue of Group 4 (medulla-contained cortex after CAM-culture). It appears that the presence of medulla in ovarian pieces is beneficial for post-thaw development of cryopreserved human ovarian tissue. For medical practice it is recommended for evaluation of post-warming ovarian tissue to use the CAM-system as a valuable alternative to xenotransplantation and for cryopreservation of these tissues to prepare ovarian medulla-contained strips.

## Introduction

Cancer is one of the major death causes: in the USA alone a total of 1,596,670 new cancer cases will be reported in 2011, of which the projected number of cancer-related deaths were 571,950. Overall cancer incidence rates in women in the USA have been declining by 0.6% annually since 1998 [1]. At the same time, the overall incidence rate for cancer in children aged 14 years and younger increased by 0.6% per year between 1998 and 2007 [2].

A similar trend has been observed in Europe. The current estimate for 2010 for Germany relates to a total of approximately 204,000 cancer cases in women, and every year in Germany, around 800 girls under age 15 are diagnosed with cancer [3]. At the same time, the death rate has been decreasing by 1.0% per year.

Over the past 25 years, there have been significant improvements in the 5-year relative survival rate for all of the major childhood cancers due to new and improved anti-cancer treatments. The 5-year relative survival rate for children with cancer improved from 58% for patients diagnosed between 1975 and 1977 to 82% for those diagnosed between 1999 and 2006 [4].

Due to the increasing of effectiveness of cancer treatments and positive long-term prognosis for young women, the problem of post-cancer infertility is playing an increasingly significant role because chemotherapy can be gonadotoxic and lead to the functional death of ovaries. Cryopreservation of ovarian tissue before cancer therapy with re-implantation after convalescence is the potential key solution of this problem [5]–[8].

Several cases reporting restoration of ovarian function after re-implantation of ovarian cortex in patients with premature ovarian failure after cancer treatment have been published since 1998 and now baby-born after thawing and transplantation is reality [9]–[31].

It is such, the final aim of the ovarian tissue cryopreservation is baby-born. However, a large body of evidence supports the notion that the primary aim of ovarian tissue cryopreservation should be for therapeutic purpose (e.g. successful restoration of ovarian function after thawing and implantation) [32], [33].

The process of preparation of ovarian tissue for cryopreservation requires the separation of cortex from medulla due to the fact that the majority of follicles are contained in the cortex. However, the discarded medulla also contains significant numbers of follicles [34] and, importantly, blood vessels. The presence of the latter may support neovascularisation in re-implanted tissues.

Part of the ovarian tissue obtained before oncological treatment is used for routine histological observation, a mandatory procedure for monitoring and minimising the risks associated with any future implantation of tissues that could be affected by metastases. After cryopreservation and storage, some ovarian tissue can be thawed and cultured in vitro in order both to screen for the presence of metastases and to monitor follicles. The quality of follicles in the cultured tissue will indicate whether it is possible to restore a woman's reproductive function. At present, there are three ways to determine effectively the quality of the cryopreservation procedure using ovarian tissue from a given patient before the re-implantation treatment: (i) evaluation of follicles after post-thawing xenotransplantation to SCID mouse [13], [35]–[39], (ii) in-vitro culture in a large volume of culture medium under constant agitation [40]–[49] and (iii) culture on embryonic chorio-allantoic membrane within a hen's egg [50]. The CAM system is an intermediate stage between in-vitro culture and animal experiments, and it could be considered as an interface between in-vitro and in-vivo models (including xenotransplantation). Importantly, it does not raise ethical or legal questions, nor does it violate animal protection laws.

The aim of this study was to compare the two methods, culture in vitro and culture on embryonic chorioallantoic membrane (CAM) of cryopreserved human ovarian medulla-containing and medulla-free cortex.

## Materials and Methods

### Ethics Statement

The protocol of investigations described in this article was approved by the Ethics Boards of Universities Cologne (permission 276-03 from 07.07.2003) and Ulm (permission 102/10 from 19.07.2010).

On the behalf of the patients under the age of 18 written consents were obtained from the next of kin.

Except where otherwise stated, all chemicals were obtained from Sigma (Sigma Chemical Co., St. Louis, MO, USA).

### Tissue collection, dissection, and distribution into groups

Informed written consent for performing of investigations was obtained from 9 patients aged between 14 and 34 (average 20.0±4.1 years). According to this protocol 10% of ovarian tissue was used for patient-oriented research.

For transportation of ovarian tissue from surgical section, preparation of ovarian strips, freezing, thawing and culture, the OvarStore™ kit (Gynemed, Lensahn, Germany) was used. This kit was developed in Universities Cologne and Ulm in cooperation with the Gynemed company. The basal medium used for manipulation of tissues (transport and dissection) was Leibovitz L-15 with 5% Dextran Serum Substitute (Irvine Scientific, Santa Ana, CA, USA), referred to below as ‘basal medium’.

Fresh ovarian tissue fragments were transported from the surgical room to the laboratory within 20 min with temperature maintained at 32–34°C. Using tweezers and scalpel No 22, ovarian fragments were dissected and divided into small pieces of two types, cortex with medulla and medulla-free cortex (1.5–2.0×1.0–1.2×0.8–1.2 mm for medulla-free pieces and 1.5–2.0×1.0–1.2×1.2–1.5 for medulla-containing pieces) and frozen as described below. After subsequent thawing (procedure described below), the pieces were randomly divided into four groups: medulla-free cortex cultured in vitro in a large volume of medium with mechanical agitation (Group 1); medulla-containing cortex cultured in vitro (Group 2); medulla-free cortex cultured in CAM-system (Group 3); medulla-containing cortex cultured in CAM-system (Group 4).

From each patient, eight pieces were used for determination of quality of follicles and angiogenesis: two pieces for each experimental group, one piece per egg.

### Freezing and thawing

The freezing protocol was based on that published by Gosden et al. [51]–[53] with some modifications. Pieces of ovarian tissue were placed at room temperature in 20 ml freezing medium (Gynemed) composed of basal medium supplemented with 6% dimethyl sulfoxide, 6% ethylene glycol and 0.15 M sucrose. Then pieces were put into a standard 1-ml cryo-straw (MTG GmbH, Bruckberg, Deutschland) previously filled by freezing medium and frozen in a CTE 2104 freezer (Cryo-Technik-Erlangen, Hoechstadt, Germany). The freezing program consisted of the following five stages: (1) start temperature 22°C, (2) cooling from 22°C to 4°C at a rate of −5°C/min, (3) cooling from 4°C to −7°C at a rate of −1°C/min, (4) cooling from −7°C to −34°C at a rate of −0.3°C/min, (5) plunging into liquid nitrogen. It was noted that at −10°C there was a formation of crystals in the place of localization of ovarian pieces (this construction of freezer implies the auto-seeding).

The procedure of thawing was achieved by holding the straws for 10 s at room temperature followed by immersion in a 100°C (boiling) water bath for 20 s, and expelling the contents of the straw into the solution for the removal of cryoprotectants. The exposure time in the boiling water was visually controlled by the presence of ice in the medium; as soon as the ice was 2 to 1 mm apex, the straw was removed from the boiling water, at which point the final temperature of the medium was between 4 and 10°C. Within 5–10 seconds after thawing, the pieces from the straw were expelled into 10 ml thawing solution (basal medium containing 0.5 M sucrose) in a 100 ml specimen container (Sarstedt, Nuembrecht, Germany). The stepwise dilution of cryoprotectants was achieved using the same principle as that used for saturation by ethylene glycol (see Fig. 1 in Isachenko et al. [40]. The container was placed on a shaker and continuously agitated with 200 osc/min for 15 min at room temperature. Stepwise rehydration of the tissue pieces for 30 min at room temperature was also performed using the same ‘dropping’ methodology [40]: slow addition of basal medium (Gynemed, see above) to the solution of sucrose with ovarian pieces. For ‘dropping’, we used 50 ml of basal medium in a 50 ml tube (Greiner Bio-One GmbH, Frickenhausen, Germany). The final sucrose concentration was 0.083 M, resulting in almost isotonic conditions. Finally, the pieces were washed thrice each in basal medium for 10 min, and transferred for in-vitro- or CAM-culture.

**Figure 1:**
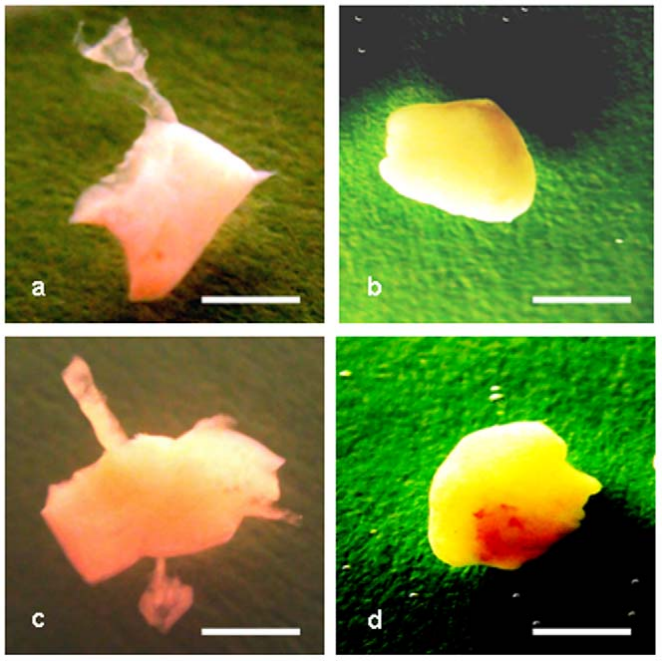
Cryopreserved ovarian medulla-free and medulla-containing ovarian pieces after thawing and 8 days in vitro culture. (a, b) medulla-free piece, (a) just after thawing, (b) the same piece after culture, (c, d) medulla-containing piece, (c) just after thawing, (d) the same piece after culture. Bar = 1 mm.

### In-vitro culture

Individual thawed pieces of two experimental groups (Fig. 1) (Group 1: medulla-free cortex and Group 2: medulla-contained cortex) were placed in 200-ml dishes for suspension culture (Cellstar, Greiner Bio-One GmbH, Germany) with 30 ml of AIM-V medium (Gibco/Invitrogen, USA). They were incubated for 8 days at 37°C in 5% CO_2_ with 75 osc/min agitation using a rotation shaker.

### CAM-culture

**Figure 2:**
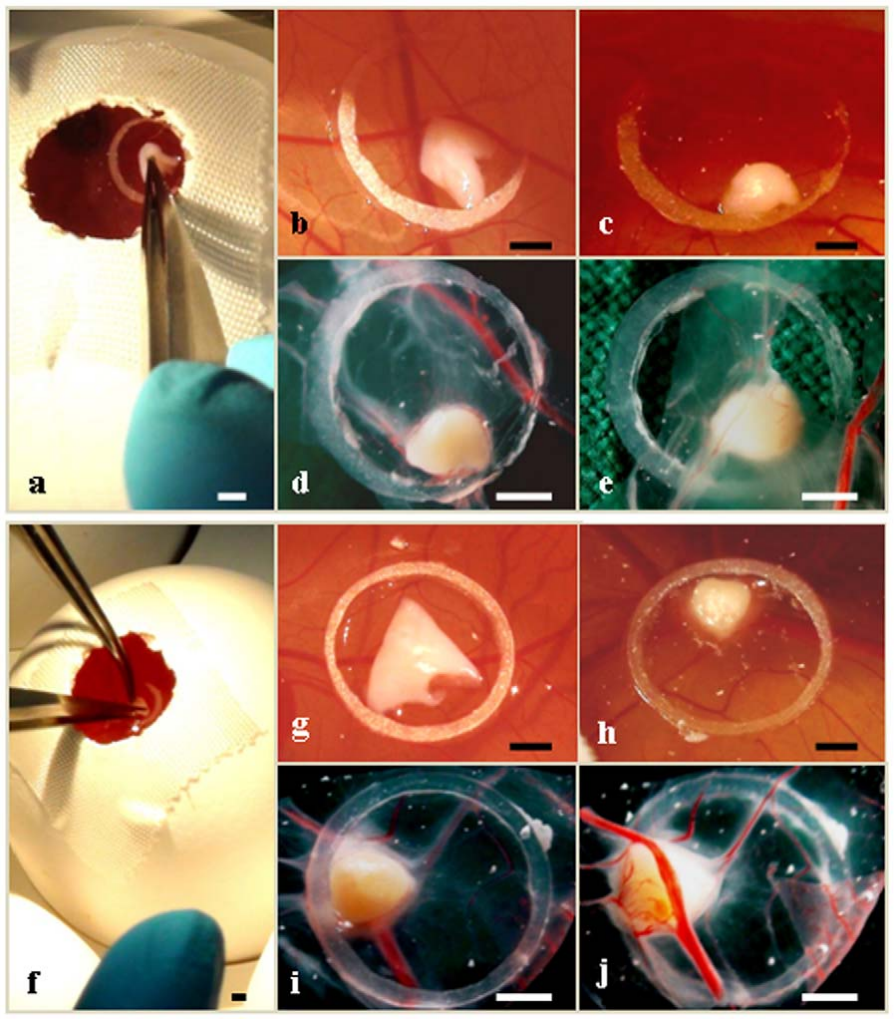
Cryopreserved ovarian medulla-free and medulla-containing pieces before and after 5 days culture with chorioallantoic membrane (CAM) system. (a, b, c, d, e) medulla-free piece, (a, b) just after thawing and seeding on CAM marked by silicone ring, (c, d, e) the same piece after culture, (c) piece on CAM, (d, e) piece in Petri dish; (f, g, h, i, j) medulla-containing piece, (f, g) just after thawing and seeding on CAM marked by silicone ring, (h, i, j) the same piece after culture, (c) piece on CAM, (d, e) piece in Petri dish; (e) outer CAM-layer with medulla-free piece, (j) inner CAM-layer with medulla-containing piece. Different intensiveness of the avian vascularisation in the place of the seeding of pieces was noted: (e) versus (j). Bar = 1 mm.

Fertilized eggs of White Leghorn chickens, purchased at a local hatchery and incubated at 37°C with 60% relative humidity, were prepared for implantation on day 4 of incubation. Standard microbiology assessment was performed to exclude subclinical infections. Preparation of the chorio-allantoic membranes was performed essentially as previously described [50], [54]–[56]. Each egg was washed with warm 70% ethanol, after which a hole was drilled through the pointed pole of the shell. The following day, part of the chorio-allantoic membrane of the embryo was exposed by peeling a 1.5–2.0 cm window in the shell. This window was covered with tape and the incubation continued. The chorio-allantantoic membrane has two epithelial layers and, in its intact form, represents a “dry” impermeable barrier for all invasions, including ovarian fragments. For “connection” of ovarian pieces with the egg system, the latter must be open. To this end, the upper peridermal part of the double epithelial layer was removed in each egg, leaving the basal layer intact. On day 10 of incubation a silicone ring 0.5 mm thick with a 5 mm inner diameter was placed on the membrane. An ovarian piece was placed into this silicone ring using microsurgical forceps (Fig. 2). Thereafter, the shell window was covered again, and the egg replaced in the incubator. After 5 days of CAM-culture the survival rate of the ovarian piece was evaluated.

### Histology of follicles

For histological investigation, the cultured tissue pieces were fixed in Bouin's solution, embedded in paraffin wax, serially sectioned at 4 µm, stained with hematoxylin/eosin, and analyzed under a microscope (×400, Olympus Co, Tokyo, Japan).

The number of viable and degenerated follicles was counted. Before counting of follicles, sections were coded and scored “blind”. To avoid overcounting of the same follicles, only the section with a visible oocyte nucleus was counted.

Morphology of follicles was evaluated on the basis of parameters previously described by Paynter *et al.* [57]. Two types of follicles were evaluated: 1) primordial follicles composed of an oocyte surrounded by a layer of flattened follicular cells and 2) primary and secondary follicles which are similar to primordial follicles, but in which the oocyte is surrounded by one to two layers of spheroid granulosa cells. The quality of follicles was graded on the scale from one to three. A follicle of grade 1 is spherical in shape and contains a spherical oocyte which is surrounded by an even distribution of granulosa cells and has a homogenous cytoplasm and slightly granulated nucleus, with condensed chromatin in the form of a dense spherical structure detectable in the center of the nucleus. A follicle of grade 2 has similar characteristics, but the oocyte is without condensed chromatin within the nucleus and is often irregular in shape; the surrounding granulosa cells can be flat and pulled away from the edge of the follicle. A follicle of grade 3 contains a misshapen oocyte with or without nuclear vacuolation; theca and granulosa cells are separated from the edge of the follicle and the partly or fully disrupted granulosa have pyknotic nuclei. Follicles of grades 1 and 2 were denoted as normal and those of grade 3 were denoted as degenerated. Examples of the different follicular degenerations can be observed elsewhere (for example, see Isachenko et al. [40], [43], [45], [47], [48].

### Immunohistology for angiogenesis

Ovarian tissue pieces after culture were fixed in 4.0% formaldehyde, embedded in paraffin wax, serially sectioned at 4 µm and stained with antibodies (Dako, Hamburg, Germany) directed against von Willebrand factor (1∶100) and desmin (1∶100).

The intensity of the immunoreactivity was semiquantitatively scored by Khan-Dawood et al. [58] as follows: lack of immunoreactivity (−), weak immunoreactivity (+), moderate immunoreactivity (++), strong immunoreactivity (+++).

In such way, the rate of vascularisation using von Willebrand factor was estimated subjectively according to the apparent surface area of blood vessels in tissue pieces, expressed as a percentage. With evaluation of neo-vascularisation, the presence of desmin expression in tissue was taken as “1”, the absence of this expression was taken as “0”.

### Statistical analysis

Post-thawing integrity rate on the parameters assessed above were evaluated by ANOVA. Normality of the distribution was checked. Various characteristics were summarized by mean and SD within groups. The level of statistical significance was set at a P<0.05.

## Results

The survival rate of the chick embryos was 97.2% (70 of 72).

After 8 days in in-vitro culture as well as 5 days after transfer onto the extra-embryonic vascular system of the chorioallantoic membrane, the ovarian fragments were observed to have developed a spherical shape (Fig. 1).

After completion of CAM-culture (on the fifth day) the propagation of ovarian pieces in the mesenchymal layer of the membrane was noted (Fig. 2). The number of distended blood vessels in the membrane near or next to the implanted ovarian fragments was markedly increased. The increased number of fine capillaries within close proximity of the implanted fragments supports the notion of their development (Figs. 2).

### Histology of follicles

**Figure 3:**
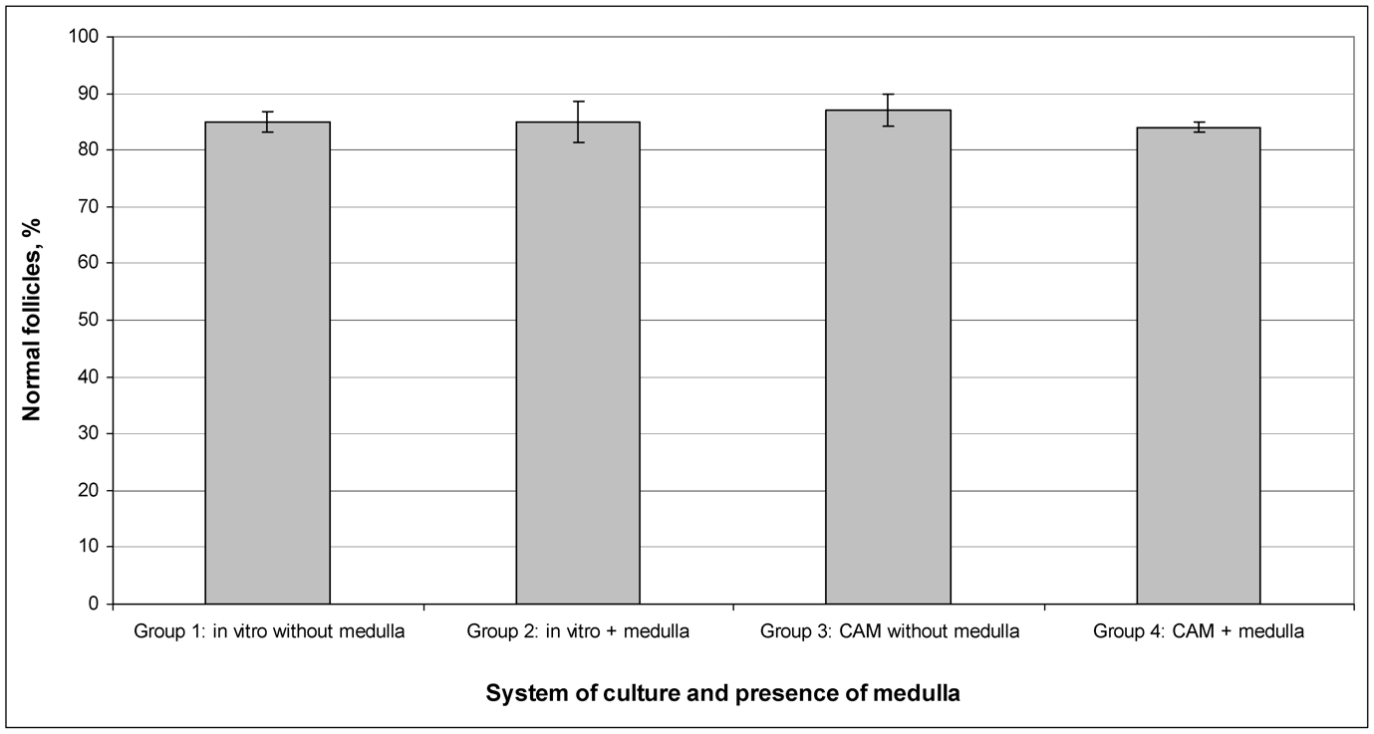
Quality of follicles (expressed as quantity and percentage of normal follicles) after in vitro and CAM-culture of cryopreserved ovarian pieces. Respective rates are not statistical different (P>0.1).

**Figure 4:**
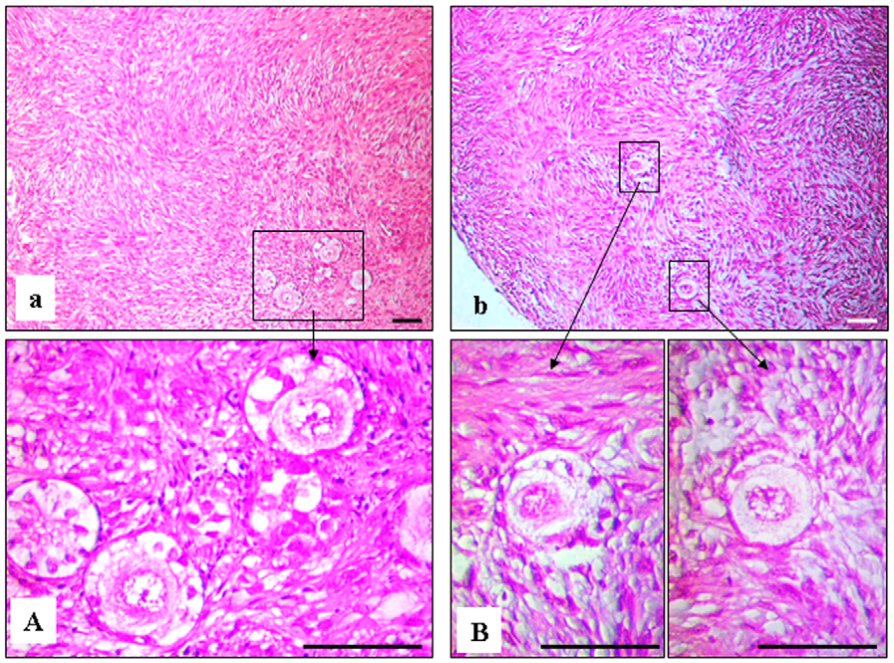
Histological micrographs of follicles from cryopreserved ovarian tissue after in vitro- and CAM-culture. (a, A) in vitro culture, (b, B) CAM culture. Bar = 25 µm.

Histological examination after the 8-day in-vitro culture and 5-day CAM-culture of ovarian pieces showed that the preantral follicles were viable. All antral follicles were degenerated after cryopreservation and culture, and were not counted. The mean preantral (primordial, primary and secondary) follicle density per 1 mm^3^ was 14.0±3.1, 12.7±2.2, 13.7±4.4, 12.0±1.2 for groups 1, 2, 3 and 4, respectively (P>0.1). Moreover, for groups 1, 2, 3 and 4 respectively, 85.0±1.8%, 85.0±3.7%, 87.0±2.8% and 84.0±0.9% of the follicles were of normal quality (grade 1 and 2) (P>0.1). There were no statistically significant differences in percentages of normal follicles between the four groups (P>0.1), thus morphological quality of follicles was independent of the conditions of culture and presence or absence of medulla (Figs. 3, 4).

### Immunohistology for angiogenesis

There were no differences detected between groups 1 and 3 (in-vitro cultured medulla-free cortex and CAM-cultured medulla-free cortex, respectively) with respect to the presence of developed blood vessels as detected with anti-vWF).

**Figure 5:**
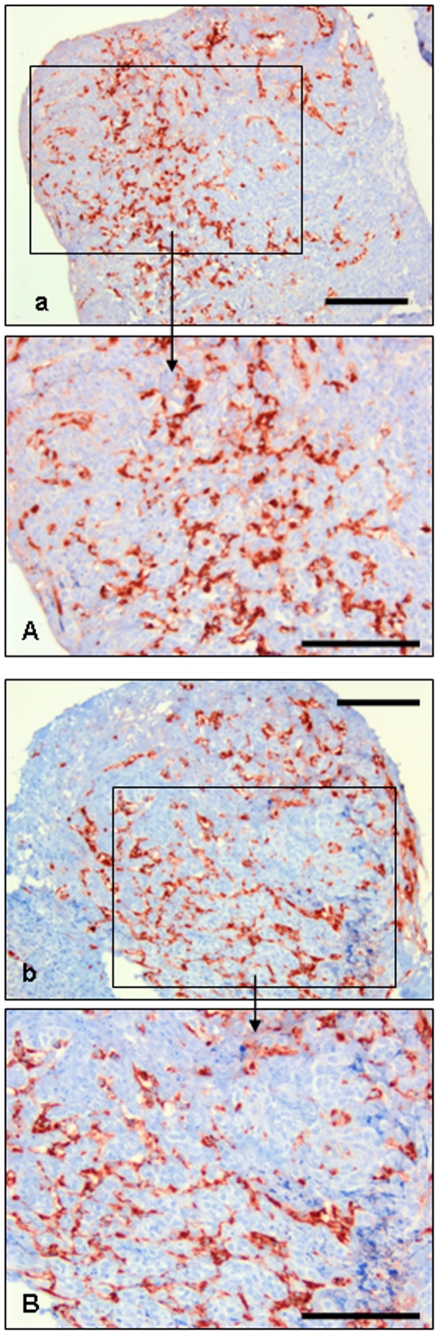
Vascularisation (von Willebrand factor expression) in cryopreserved medulla-containing ovarian tissue after in vitro and CAM-culture. Immonostained after in vitro (a, A) and CAM (b, B) culture. Bar = 0.25 mm.

Figure 5 shows representative appearances of vascularisation in frozen-thawed tissues.

The following rates of this immunoreactivity were observed:

Group 1 (medulla-free cortex cultured in vitro): weak (+);

Group 2 (medulla-containing cortex cultured in vitro): moderate (++);

Group 3 (medulla-free cortex cultured in CAM-system): moderate (++);

Group 4 (medulla-containing cortex cultured in CAM-system): strong (+++).

In groups 1 and 3, the degree of vascularisation (as estimated subjectively according to the surface area occupied by the blood vessels) was about 40%, whereas this rate was about 70% in groups 2 and 4 (in-vitro cultured medulla-containing cortex and CAM-cultured medulla-containing cortex, respectively) (P<0.05) (Fig. 5).

Figure 5 shows representative appearances of vascularisation in frozen-thawed tissues.

**Figure 6:**
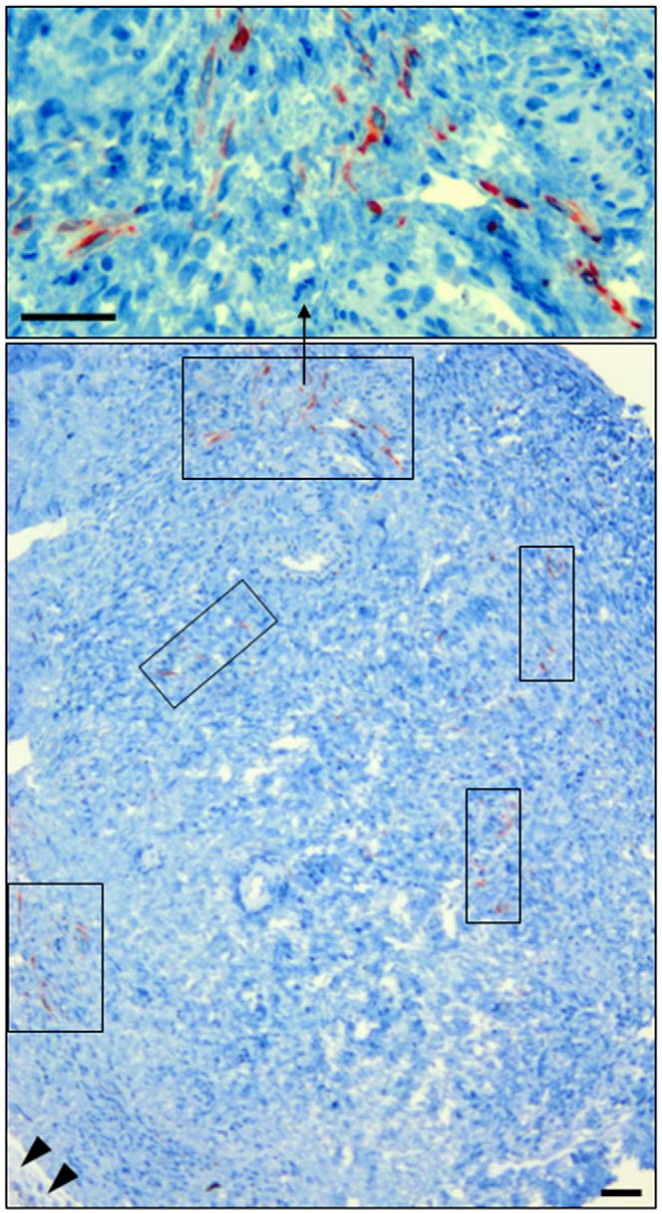
Neo-angiogenesis (desmin expression) in cryopreserved medulla-containing ovarian tissue after CAM-culture. (rectangles) places where neo-angiogenesis was detected, (head arrow) CAM. Bar = 25 µm.

Neo-angiogenesis (detected with anti-desmin) was observed only in Group 4 (CAM-cultured medulla-contained cortex) (Fig. 6). In tissue of groups 1, 2 and 3 this process was not observed.

## Discussion

The object of this study was two-fold. Firstly we wished to compare two methods of culture, in vitro-culture.and culture on the embryonic chorioallantois membrane (CAM) of fertilized chicken eggs as a means of assessing the quality of cryopreserved human ovarian tissue [50]. Secondly we wished to compare during culture the survival of cryopreserved medulla-containing tissue with that of medulla-free tissue.

The chorioallantoic membrane system is a simple inexpensive method. The extraembryonic vessel system of the chick chorioallantois membrane is naturally immunodeficient, similar to the mammalian placenta which is also not innervated. Moreover the embryonic neural and immune systems have not yet developed in the first half of the normal chick development period. Because of these special characteristics, the CAM system can be used for xenotransplantation of different types of cells from different species.

The CAM-System is used to study angiogenesis in tumour [59] and endometriosis tissues [60]. CAM also been used for culture of human skin [55], liver [61] and skeletal muscle tissue [62], for surgical retinal research [63], for testing of different biomaterials in tissue engineering [64] and also for investigations of a bright spectrum on biological objects [65].

In CAM-culture of bovine and murine ovarian tissue, reported by Cushman et al. [66] and Gigli et al. [67] respectively, primordial follicles in the transplanted ovarian tissue retained the ability to activate and grow. These authors believe that the extracellular matrix constitution of CAM is similar to the peritoneum, to which human ovarian tissue can be transplanted via intraperitoneal xenotransplantation and orthotopic autotransplantation.

Our results indicate that the morphological quality of follicles was independent of the conditions of culture and of the presence or absence of medulla. Thus the CAM system is not disadvantageous in this respect.

The first stage in ovarian tissue cryopreservation has usually been the isolation of cortex from medulla [7]. This procedure allows the cryopreservation of primordial follicles, which are small and cryo-resistant structures [68], [69]. Usually 1–2 mm slices of cortex are prepared without medulla.

However, the ovarian medulla has two crucial characteristics: the presence of follicles and the presence of blood vessels. Follicle density in the medulla can reach 9824 follicles/gram of medulla, and considerable numbers of pre-antral follicles are lost when discarding the medulla during the current practice of isolating the cortical tissue for cryopreservation [34].

Why medulla-mediated promotion of neovascularization is advantageous for re-implanted ovarian cortex?

What advantage neo-vascularization is going to add to the preservation and/or development of follicles after transplantation?

The presence of blood vessels is a very important factor for successful ovarian graft transplantation as it is required for the rapid establishment of the blood supplies crucial for the survival of ovarian follicles [70]. It was showed that transplanted immature rat ovaries become profusely re-vascularised within 48 h after autotransplantation [71]. In the cortex, development of primordial follicles are fully dependent on stromal vessels [72]. Prior to re-vascularisation, implants are vulnerable to ischaemia, which is the main obstacle for the survival of tissue after transplantation. Such damage can lead to a 30 to 70% reduction in graft size accompanied with fibrotic changes [73]. The hypoxia observed during the first 5 days after grafting and ischaemic damage occurring during this period could induce primordial follicle loss [74]–[76] and disorders of follicular activation [11], [77].

The presence of medullary material in ovarian cortex pieces could be expected to improve the chances of revascularization of transplanted ovarian tissue. In examining the histology of our cultured ovarian fragments, we therefore sought expression of Von Willebrand factor and desmin.

Von Willebrand factor (vWF or factor VIII-related antigen) is a blood adhesive and multimeric glycoprotein [78] involved in hemostasis [79], which mediates platelet adhesion to sub-endothelium at sites of vascular injury and binds and stabilizes factor VIII in the circulation [80], [81]. It is synthesized in endothelial cells [82]. Most recently, it was hypothesized that vWF regulates angiogenesis [83]. However, in immunohistochemistry, the staining intensity of vWF is dependent on the blood vessel size; the most prominent expression of vWF is displayed in veins, followed by arteries, arterioles, capillaries, and venules [84], [85].

In fact, neo-vascularization begins with the formation of very small vessels. Thus, to detect the formation of these vessels we have used desmin. Desmin is presenting during the initial development of vessel sprouts and exhibits differential staining patterns during vessel formation [86]. Pericytes, connective tissue cells that occurs about small blood vessels [87], appear to be involved in the earliest stages of capillary sprouting and were regularly found lying at and in front of the advancing tips of endothelial sprouts. It was investigated the participation of microvascular pericytes in the process of capillary sprouting. Capillary sprouts were visualized by staining with rhodamine-conjugated phalloidin and pericytes were simultaneously stained by an antibody to desmin. It was established that developing pericytes were clearly reactive for desmin [88].

The presence of desmin-positive cells at the early stage of vascularization can be shown by the following results [89]. The angiogenesis induced after implantation of carcinoma fragments was investigated. The vascular development, which was clearly concentrated in a dense rim around the tumour, suggests an acquisition of vasculature by the tumour through vessel incorporation. Initially, scattered desmin-positive cells were found in the developing angiogenic rim. Later many desmin-positive cells, which were identified by electron microscopy as pericytes, were found around vessels and exhibited close local contacts with endothelial cells. Later however, after incorporation of the peritumour vascular rim into the tumour, the number of pericytes decreased [89].

Our subjective observations support the notion that the rate of vascularisation (detected with anti-vWF) was higher in medulla-containing cortex than in medulla-free cortex, regardless of culture system. Moreover, we observed neo-angiogenesis (detected with anti-desmin) only in CAM-cultured medulla-containing cortex.

Overall, our preliminary results as presented here lead to three conclusions. Firstly, they support the observation by Martinez-Madrid et al. [50] that the CAM system can be used for culturing cryopreserved human ovarian tissue. Secondly, it appears that the presence of medullar material in the ovarian pieces leads to better vascularization and is crucially needed for neo-angiogenesis; it can be inferred that inclusion of medulla would be of important advantage when such pieces are re-implanted into their female sources. Thirdly, only culture in the CAM system led to neo-angiogenesis in the medulla-containing pieces. For medical practice it is recommended for evaluation of post-warming ovarian tissue to use the CAM-system as a valuable alternative to xenotransplantation and for cryopreservation of these tissues to prepare ovarian medulla-contained strips.
